# Selenium Supplementation and Crop Plant Tolerance to Metal/Metalloid Toxicity

**DOI:** 10.3389/fpls.2021.792770

**Published:** 2022-01-03

**Authors:** Mirza Hasanuzzaman, Kamrun Nahar, Pedro García-Caparrós, Khursheda Parvin, Faisal Zulfiqar, Naznin Ahmed, Masayuki Fujita

**Affiliations:** ^1^Department of Agronomy, Faculty of Agriculture, Sher-e-Bangla Agricultural University, Dhaka, Bangladesh; ^2^Department of Agricultural Botany, Faculty of Agriculture, Sher-e-Bangla Agricultural University, Dhaka, Bangladesh; ^3^Agronomy Department of Superior School Engineering, University of Almería, Almería, Spain; ^4^Department of Horticulture, Faculty of Agriculture, Sher-e-Bangla Agricultural University, Dhaka, Bangladesh; ^5^Department of Horticultural Sciences, Faculty of Agriculture and Environment, The Islamia University of Bahawalpur, Bahawalpur, Pakistan; ^6^Independent Researcher, Dhaka, Bangladesh; ^7^Department of Applied Biological Science, Faculty of Agriculture, Kagawa University, Kagawa, Japan

**Keywords:** abiotic stress, biofortification, heavy metals, phytoremediation, plant nutrition, oxidative stress, trace elements, xenobiotics

## Abstract

Selenium (Se) supplementation can restrict metal uptake by roots and translocation to shoots, which is one of the vital stress tolerance mechanisms. Selenium can also enhance cellular functions like membrane stability, mineral nutrition homeostasis, antioxidant response, photosynthesis, and thus improve plant growth and development under metal/metalloid stress. Metal/metalloid toxicity decreases crop productivity and uptake of metal/metalloid through food chain causes health hazards. Selenium has been recognized as an element essential for the functioning of the human physiology and is a beneficial element for plants. Low concentrations of Se can mitigate metal/metalloid toxicity in plants and improve tolerance in various ways. Selenium stimulates the biosynthesis of hormones for remodeling the root architecture that decreases metal uptake. Growth enhancing function of Se has been reported in a number of studies, which is the outcome of improvement of various physiological features. Photosynthesis has been improved by Se supplementation under metal/metalloid stress due to the prevention of pigment destruction, sustained enzymatic activity, improved stomatal function, and photosystem activity. By modulating the antioxidant defense system Se mitigates oxidative stress. Selenium improves the yield and quality of plants. However, excessive concentration of Se exerts toxic effects on plants. This review presents the role of Se for improving plant tolerance to metal/metalloid stress.

## Introduction

As plants and environments are intensively connected, they face multifaceted stresses due to their sessile nature. Due to rapid industrialization and agricultural development, plenty of wastewater, fertilizers, and pesticides are discharged that result in toxic metal/metalloid contamination of soil and other environmental components (Feng et al., 2020). Exposure of plants to various metals including cadmium (Cd), chromium (Cr), lead (Pb), arsenic (As), copper (Cu), nickel (Ni), iron (Fe), mercury (Hg), antimony (Sb) etc. causes the alteration of physiological and biochemical processes by higher metal uptake and translocation, thereby hampering the normal growth and development of the plant. In addition, these unrest the cellular metabolic functions by binding with proteins, nucleic acids, and enzymes (Hasanuzzaman et al., 2019). The obvious effect of metal stress is the stimulation of reactive oxygen species (ROS) generation, which causes the oxidative stress by damaging cellular macromolecules including lipids, protein, and DNA. The ultimate extreme levels of metal/metalloid can lead to plant death (Hasanuzzaman et al., 2020). Contrarily, plants possess the defense tactics for keeping ROS at a nontoxic level to regulate its signaling roles through involving non-enzymatic and enzymatic antioxidants (Hasanuzzaman et al., 2019). Toxic metal contamination in the environment also causes threats to human health *via* the food chain (Feng et al., 2020).

Therefore, much attention should be given for inducing higher metal stress tolerances of plants for increasing worldwide crop production along with reducing health hazard. For aiming this, thirst of researchers is to develop technology or management practice, which will help to reduce metal uptake in plants in contaminated soil. Consequently, the use of chemical approaches, especially essential micronutrients like selenium (Se), has become one of the effective strategies to mitigate the toxic effects of metals because of its biochemical functions (Wang et al., 2020). It is beneficial in regulating plant growth and development by alleviating environmental stress-induced damage when applied at a low concentration (Ismael et al., 2019). Particularly, Se supplementation can restrict metal absorption by roots and translocation to shoots, which is one of the vital metals/metalloid stress tolerance mechanism (Hasanuzzaman et al., 2020). Besides, Se-induced improvement in cellular functions and membrane stability, mineral nutrition with upregulation of antioxidants response, and metabolites function and also reduction of oxidative stress in plants has been widely explored against metal stresses (Gupta and Gupta, 2017; Zhao et al., 2019; Wang et al., 2020). Such roles of Se at low dose in plant growth as antioxidants, stress alleviators, and uptake inhibitor of metals, including Cd (Luo et al., 2019), Cr (Ulhassan et al., 2019), Pb (Wu et al., 2016), As (Shahid et al., 2019), Cu (Trevisan et al., 2011), Hg (Tran et al., 2018), etc. have been reported already. Additionally, lower Se concentration is able to stimulate the biosynthesis of hormone-like auxin, which causes the remodeling of root architecture with higher root growth for resulting in lower metal uptake (Feng et al., 2020). Supplementation of Se causes an increase of pectin and hemicellulose contents as well as thickness of cell wall, which enhances the binding of toxic metals by the cell wall (Zhao et al., 2019). Selenium can regulate the subcellular distribution of metals. Moreover, exogenous Se application in various major crops including rice, lettuce, cucumber, *Brassica*, etc. significantly has been reported to reduce metal accumulation with growth improvement, which also ensures better crop productivity and health benefits to consumers (Pandey and Gupta, 2018). It is also reported that, excessive concentration of Se is also toxic for plants by causing chlorosis, growth reduction, and even oxidative stress (Molnár et al., 2018; Hasanuzzaman et al., 2020); that is why it is imperative to emphasize selecting the optimal dose of Se. Therefore, extensive and broad scale research is still required for determining the crucial dose and engagements of Se for attaining plant tolerance toward metals. In this review, information on the potentiality of Se for reducing metal toxicity in plants through various strategies, including restriction of metal uptake, regulation of ROS metabolism, responses of antioxidants, and ion homeostasis, which are associated with the improvement of plant physiology, growth, development, and yield has been gathered, which will be supportive to get insight into Se-induced metal tolerance in plants.

## Plant Responses to Metal/Metalloid Toxicity

All the metals/ metalloids, either essential or nonessential, produce toxic effects on plants, which result in poor biomass accumulation and stunted growth whenever they present above their threshold levels. In *Zea mays*, Cd (CdSO_4_, 6 mg kg^−1^) phytotoxicity decreased plant height and above ground fresh weight (FW) by 21 and 22% compared with control (Liu et al., 2018). Upon exposure to Cd stress, growth attributes of *Brassica oleracea* including root FW, root dry weight (DW), shoot FW, and shoot DW decreased by 14, 41, 27, and 53%, respectively over the control due to the accumulation of Cd in plant root and shoot (Shah et al., 2020). Combination of Cd (100 μM, CdCl_2_) and As (150 μM, NaAsO_2_) reduced plant height, leaf area, shoot FW, and shoot DW in two cultivar of *Z. mays*. Among two cultivars, Dong Dan 80 showed higher tolerance to Cd and As stress compared with Run Nong 35 (Anjum et al., 2017). In *Cicer arietinum*, reduction of plant height (by 46%), plant FW (by 90%), plant DW (by 89%), number of primary branches (by 80%), and secondary branches (by 84%) were observed under Cr (120 μM, K_2_Cr_2_O_7_) over the control (Singh et al., 2020).

Metal toxicity led to several deleterious effects on physiological attributes of plants including chlorophyll (chl) functioning, stomatal conductance (g_*s*_), net photosynthetic rate (P_n_), intercellular CO_2_ concentration (C_i_), transpiration rate (T_r_), and photosynthetic enzyme activities (Table 1). Shah et al. (2020) stated that water potential and leaf osmotic potential in *B. oleracea* decreased in plants subjected to Cd stress and resulted in a sharp decline of leaf relative water content (RWC) by 46% in comparison with control condition. Cadmium toxicity along with leaf-Fe deficiency affects photosynthetic electron transport and reduced CO_2_ fixation, maximal and actual efficiency of photosystem II (PS II), chl synthesis, etc. (Lešková et al., 2017). Reduction of C_i_ (by 20.33%), g_*s*_ (by 39%), and T_r_ (by 10%) under Cd stress was observed in *B. oleracea* (Shah et al., 2020).

**Table 1:** Influence of metal/metalloid toxicity on different plants.

**Species**	**Metal/metalloid treatment**	**Major effects**	**References**
*Zea mays*	Cd (3 and 6 mg kg^−1^ CdSO_4_), 60 d	Reduced plant height, plant fresh weight (FW)	Liu et al., 2018
*Brassica oleracea*	Cd (5 mg L^−1^), 28 d	Reduced growth attributes, photosynthetic pigments, intercellular CO_2_ concentration (C_i_), stomatal conductance (g*_*s*_*) and transpiration rate (T_r_)	Shah et al., 2020
*Triticum aestivum*	Cd (100 μM CdCl_2_), 10 d	Declined leaf water potential, chlorophyll (chl) content and maximum quantum yield (F_v_/F_m_)	Kaya et al., 2019
*Fragaria ananassa*	Cd (80 mg L^−1^, CdCl_2_) 7 d	Decreased plant height and plant biomass, chl and carotenoid content, average fruit weight, vitamin C and soluble sugar content	Zhang et al., 2020
*Z. mays*	As (150 μM NaAsO_2_), 15 d	Reduction of plant height, stem diameter, shoot FW, shoot DW, no. of leaves plant^−1^, leaf area, net photosynthetic rate (P_n)_, g_s_ and T_r_; C_i_, grain yield and 100 grain weight	Anjum et al., 2017
*T. aestivum*	As (50 and 100 ppm), 84 d	Reduced growth attributes, water use efficiency, C_i_, g_s_, T_r_, chl, flavonoid and anthocyanin content	Ali and Perveen, 2020
*Oryza sativa*	As (25 μM Na_3_AsO_4_), 10 d	Restriction of plant growth with higher chlorosis, membrane damage, production of H_2_O_2_ and malondialdehyde (MDA)	Banerjee et al., 2020
*Vicia faba*	As (5 μM Na_3_AsO_4_), 27 d	Inhibition of chl biosynthesis with higher chlorophyllase activity, gas exchange parameters including P_n_, g_s_ and C_i_ as well as elevated reactive oxygen species (ROS) accumulation	Siddiqui et al., 2020
*V. faba*	As (5 μM Na_3_AsO_4_), 30 d	Decrease of chl content with higher chlorophyllase activity, electrolyte leakage, ROS accumulation, nitrate reductase and nitrite reductase activity	Siddiqui et al., 2021
*Solanum melongena*	As (25 μM Na_3_AsO_4_), 17 d	Reduction in seedling fresh and dry weight, root growth and P uptake, F_v_/F_m_, photosynthesis with higher ROS and cell death	Alamri et al., 2021
*C. arietinum*	Cr (30, 60, 90, 120 μM, K_2_Cr_2_O_7_), 72 h	Reduced germination, germination index, root length and shoot length	Singh et al., 2020
*B. Juncea*	Ni (100, 200 and 400 μM NiSO_4_.6 H_2_O), 7d	Reduced seed germination, root length, shoot length and seedling FW	Thakur and Sharma, 2016
*O. sativa*	Ni (50 and 200 μM NiSO_4_.6 H_2_O), 9 d	Reduction of root length, shoot length, seedling FW and seedling DW and soluble protein	Rizwan et al., 2018
*B. Juncea*	Ni (50 and 100, 150 μM of NiCl_2_.6 H_2_O), 14 d	Reduction of root length, shoot length, plant DW, leaf relative water content (RWC), total chl and carotenoid content, T_r_, C_i_ and increased g*_*s*_*	Abd_Allah et al., 2019
*Solanum melongena*	Ni (100 mg kg^−1^ of NiCl_2_. 6H_2_O), 14 d	Reduction of growth, leaf water status, pigment content, g_s_, ci and T_r_	Shah et al., 2021
*S. lycopersicum*	Cu (10 and 100 mg kg^−1^ of CuSO_4_·5H_2_O), 40 d	Alterations in root morphology, reduced chl content and photosynthetic capacity and stomata aperture	Nazir et al., 2019
*O. sativa*	Pb (1,200 mg kg^−1^ of Pb(NO_3_)_2_), 95 d	Reduction in pigments content, decreased filled grain percentage, grain yield and harvest index	Ashraf and Tang, 2017
*T. aestivum*	Pb (0.5 and 1 mM of Pb (NO_3_)_2_), 48 h	Reduction in plant height, FW and DW, leaf water status and pigment concentrations	Hasanuzzaman et al., 2018
*Helianthus annuus*	Pb (300, 600 and 900 mg kg^−1^, PbNO_3_), 105 d	Reduced root and shoot FW and DW, chl *a*, chl *b* and carotenoid content and yield plant^−1^	Saleem et al., 2018
*C. arietinum*	Hg (15, and 30 μM) HgCl_2_, 70 d	Reduction in root and shoot growth, reduced pigment concentration and leaf RWC	Ahmad et al., 2018
*H. tuberosus*	Hg (0.15, 1, 5 and 10 mg kg^−1^ of HgCl_2_), 70 d	Delay in seedling time emergence, reduction of leaf area and growth and chl content	Lv et al., 2018
*Allium sativum*	Hg (3 mg L^−1^ HgCl_2_), 185 d	Decreased growth attributes, P_n_, g*_*s*_* and T_r_	Hu Y. et al., 2020

Toxic metals reduce CO_2_ fixation by downregulating the activity of ribulose-1,5-bisphosphatecarboxylase-oxygenase (RuBisCO) or by reacting with its thiol group. In Cd-treated (100 and 200 μM CdCl_2_) plants, chloroplast structure is damaged due to the increased production of small starch grain, enlarged osmiophilic globules which led to condensation of grana and lamella (Guo et al., 2016). Reduction of P_n_, T_r_, g_*s*_, and chl synthesis was higher in combined application of Cd and As treatments in sensitive cultivar Dong Dan 80 compared with more tolerant Run Nong 35 (Anjum et al., 2017). Nitrogen metabolism (N uptake, transfer, and assimilation) is also affected by toxic metals/metalloids due to the altered activity of N-assimilatory enzymes. Combined application of Cd (40 μM CdCl_2_) and As (40 μM Na_2_HAsO_4_. 7H_2_O) in *Solanum tuberosum* significantly inhibited nitrate reductase (NR) and nitrite reductase (NiR) activities of leaves, roots, and stolons (Shahid et al., 2019). Metals also downregulated ammonia assimilation through reducing the activity of enzymes, namely glutamine, glutamate synthetase, and glutamate dehydrogenase (Hussain et al., 2020).

Under high metal/metalloids concentration, there is an increase in the synthesis of ROS such as superoxide (${\text{O}}_{2}^{•-}$) and hydroxyl (OH^•^) free radicals, or non-free radical species such as singlet oxygen (^1^O_2_) and hydrogen peroxide (H_2_O_2_). Methylglyoxal (MG) with a high cytotoxicity is also generated resulting in an imbalance in the antioxidant cell homeostasis. Consequently, the imbalance led to the lipids and protein oxidation, ion leakage, DNA injuries, and even the programmed cell death (Syed et al., 2018).

**Figure 1:**
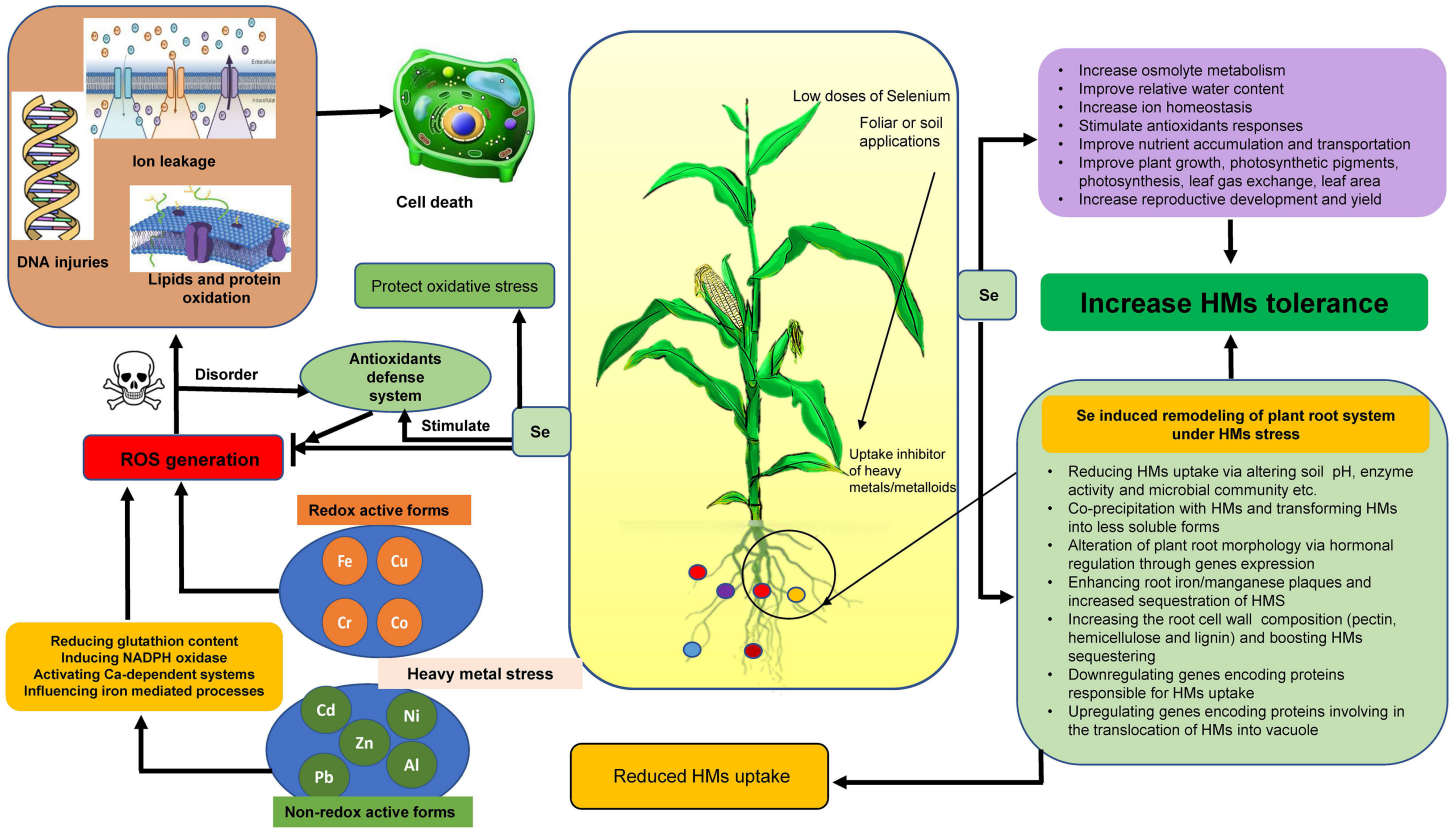
ROS generation under heavy metal stress and protective roles of selenium.

The exposure of plants to metal/metalloids causes oxidative stress which can affect the cellular homeostasis in different ways (i) inducing the generation of ROS, (ii) direct generation of ROS *via* Fenton like reactions and the Haber–Weiss cycle, and (iii) consumption of element of cellular redox homeostasis such as glutathione as direct chelator and/or as a precursor of phytochelatines (Sharma and Dietz, 2009). Metal/metalloids such as Cu, Cr, Fe, and Co are redox active, being able to generate ROS directly *via* Haber–Weiss and Fenton reactions, whereas Zn, Ni, Cd, Pb, and Al are non-redox reactive and participate in the synthesis of ROS through the reduction of glutathione content, promoting NADPH oxidase activity or with the initiation of calcium-dependent systems and participating in iron-mediated processes (Figure 1; Mahmud et al., 2018).

Metal/metalloid toxicity alters the physiological processes, which leads to stunted growth, reduced biomass, and yield loss. In *Triticum aestivum*, stress resulted in significant reduction of grain yield plant^−1^, no. of seeds plant^−1^, and 100 grain weight (Ali and Perveen, 2020). Responses of *Oryza sativa* to Cd toxicity depends on the storage of Cd in root and translocation through the root to stem and grain, stress doses, and cultivars variation. Different Cd levels (CdCl_2_; 50, 100 and 150 mg kg^−1^) were applied on five cultivars of *O. sativa*, and their effects on yield were studied as compared with control where grain yield reduced by 34, 43, and 64% for Meixianzhang-2, 10, 31, and 39% for Xiangyaxianzhang; 11, 24, and 40% for Guixianzhang; 12, 23, and 31% for Basmati, and 29, 46, and 48% for Nongxian 18, respectively. Highest panicle numbers, spikelet panicle^−1^, 1,000-grain weight, seed setting, and grain yield were found in Guixiangzhan, followed by Meixianzhang-2, whereas lowest values of these parameters were observed in Nongxian 18 and Xiangyaxiangzhan (Kanu et al., 2017). Quality attributes of *O. sativa* including brown rice rate, milled rice rate, milling degree, head rice rate, moisture content, protein content, and 2-acetyl-1-pyrroline content decreased, whereas the chalkiness rate and chalkiness degree increased significantly under Cd (CdCl_2_, 100 mg kg^−1^) stress due to the increased oxidative damage (Imran et al., 2021). Exposure to Cd stress (80 mg L^−1^, CdCl_2_) inhibited growth with increased accumulation of Cd in leaf and fruit of strawberry; thus, reducing average fruit weight, vitamin C, and soluble sugar content in the fruit (Zhang et al., 2020). Application of 120 μM K_2_Cr_2_O_7_ in *C. arietinum* significantly reduced yield and different yield-contributing parameters, including number of pods plant^−1^ and number of seeds pod^−1^ (Singh et al., 2020).

## Protective Role of Selenium in Plant

The progress in unveiling the protective roles of Se in plants shows that Se can act as a vital component for plant growth, development, and induce tolerance toward abiotic stresses in the era of climate change (Hasanuzzaman et al., 2020). Selenium is reported to play a vital role in mitigation of metal stress (Figure 1; Feng et al., 2013). Selenium protects plants from toxic metals by reducing their uptake and transportation from roots to upper plant parts like shoots, leaves, and even grain (Gao et al., 2018). The excessive production of ROS is reported to decrease with Se supplementation by boosting antioxidant defense system under stress (Hasanuzzaman et al., 2020). Moreover, soil or foliar Se treatment (50 g ha^−1^) boosted chl formation and photosynthesis and also increased the antioxidant capacity as well as the yield of cowpea (*Vigna unguiculata* L. Walp.) plants (Silva et al., 2018). In another work, Se application under normal conditions to quinoa improved growth, photosynthesis, proline (Pro), antioxidants, and reduced the ROS production (Khalofah et al., 2021). Selenium application also reported to influence osmolytes under various abiotic stresses. For instance, Khan et al. (2015) observed a decline in Cd-induced oxidative stress in wheat *via* altering Pro and glutathione production. In another work, Regni et al. (2021) reported a stabilized level of Pro in the leaves and a reduced loss of this osmolyte from the roots under salt stress in olive plants. Selenium application to plants is also observed to enhance nutrient uptake in plants under abiotic stresses. For instance, in salt-stressed bread wheat, application of Se (8 μM) improved Ca and K concentration and improved salt stress tolerance (Desoky et al., 2021). Selenium nanoparticles are also reported to play an important role in ameliorating the Cd-induced oxidative stress in *B. napus* (Qi et al., 2021). However, the high doses of Se causes phytotoxicity symptoms, such as cell plasma membrane disruption, chlorosis, senescence, and reduced yield (Ribeiro et al., 2016; Silva et al., 2018).

## Role of Se in Metal/Metalloid Tolerance in Plants

### Se Minimizes Metal/Metalloids Uptake

Selenium causes the restriction of toxic metal ions uptake and their metabolism by rescuing their translocation from lower plant to upper parts (Alyemeni et al., 2018; Hasanuzzaman et al., 2020). For instance, such Se-induced lower Cd uptake by roots had been reported in Cd-stressed tomato seedlings along with reduced translocation into shoots and leaves (Alyemeni et al., 2018). Not only that, Se alters the translocation ability of cultivar through lowering root to stem and stem to grain along with increasing stem to leaves translocation of different rice cultivars (Gao et al., 2018). Supplemented Se is able to enhance the binding of metal ions to the root cell wall by improving pectin and hemicellulose, and also changes the subcellular distribution in roots, for example metal contents are reduced in plastids and mitochondria, whereas enhanced in vacuolar sap and ribosome (Zhao et al., 2019). Moreover, protective role of Se is also involved in improving phytochelatin (PC) synthesis or nontoxic Se-metal complexes formation as well as reducing metals transportation from root to aerial plant parts for alleviating metal toxicity (Hawrylak-Nowak et al., 2014).

In *B. campestris* sp. *Pekinensis*, exogenous application of Se reduced the concentration and influx of Cr by altering root morphology (Zhao et al., 2019). Ulhassan et al. (2019) observed a decrease in Cr accumulation in plant tissues with the application of Se in *B. napus*. Hawrylak-Nowak et al. (2014) reported decrease in Cd accumulation and phytochelatins in roots of cucumber and an increased translocation toward shoot in Se-treated plants. Conversely, some studies have demonstrated that Se supplementation has no significant impact in the accumulation of Pb in the bean grown under field conditions (Mroczek-Zdyrska and Wójcik, 2012; Mroczek-Zdyrska et al., 2017). Pokhrel et al. (2020) reported a decrease in As uptake in rice. Se application influences the uptake of heavy metals in a time-dependent manner. For instance, in rice grown under Cd stress, supplementation of Se enhanced the uptake of Cd, but decreased this uptake on longer Cd exposure (Wan et al., 2016). Further Se-induced decrease in heavy metal uptake in plant varies even among the cultivars of the plant species. Gao et al. (2018) evaluated the influence of Si and Se individually and in combination on the uptake of Cd in three rice cultivars namely WYHZ, NJ5055, and ZF1Y. They observed that all the three forms of protectants applications (Si, Se, and Si + Se) decreased the uptake of Cd in WYHZ, but such influence was not noted in NJ5055 and ZF1Y.

### Se Improves Plant Growth and Physiology Under Metal/Metalloid Toxicity

Selenium plays a crucial role in the protection of plants from the toxic impacts of metals/metalloids (Feng et al., 2020; Riaz et al., 2021). It is a common phenomenon that plants under the exposure of metals/metalloids tend to reduce their growth. A number of studies have unveiled that Se in different forms applied at different levels to crop plants grown under heavy metals/metalloids stress boost plant growth and improve physiological functions. Hawrylak-Nowak and Matraszek-Gawron (2020) evaluated the influence of 2 or 6 μM of different forms of Se (selenite IV and selenate VI applied as Na_2_SeO_3_ or Na_2_SeO_4_) on Ni (5 and 10 μM)-exposed lettuce plants. The authors observed that Se (IV) at 2 or 6 μM levels caused a reduction in leaf area, shoot (34 and 72%), and root (39 and 73%) FW under 5 μM Ni, whereas a decrease of 82–84% in shoot and root FW was observed in 6 μM Se (IV)-supplemented plants grown under 10 μM Ni. Contrary to this, 2 μM Se(VI) did not influence leaf area and biomass but improved root biomass (23%) under 10 μM Ni stress. Regarding physiological impacts, application of Se (IV) and (VI) improved photosynthetic pigments, except 6 μM Se (IV) (Hawrylak-Nowak and Matraszek-Gawron, 2020). In *B. juncea*, application of selenite (Se IV, 50 μM) not only enhanced germination by improving germination rate and seedling vigor, but also improved growth by reducing As-induced (As III, 300 μM) adverse effects in shoot and root length, fresh and dry weight by about 9, 12, 5, and 9%, respectively with improvement of root/shoot ratio by 9% under (Sahay et al., 2020). In another work, Handa et al. (2019) reported that in *B. juncea* grown under Cr (300 μM kg^−1^), application of Se (4 μM kg^−1^) recovered root and shoot length by 44 and 18%, respectively. Nano-Se is also reported to provide a relieving effect to crop plants grown in contaminated soils. For instance, supplementation of nano-Se to rice plants grown under Pb and Cd contaminated soil improved plant growth and photosynthesis and its related genes, proteins, and chl concentration (Wang et al., 2021). Alves et al. (2020) also reported that application of 1.0 μM of selenite or selenate resulted in an increase in photosynthesis and biomass of tomato grown under Cd stress (0.5 mM CdCl_2_). In wheat, combined application of Se and Zn (10, 20, and 40 mg L^−1^) alleviated the Cd-induced reduction in growth, photosynthesis, and photosynthetic pigments (Wu et al., 2020). Han et al. (2015) reported an increase in growth of *Nicotiana tabacum* under As stress (0–5 mg L^−1^) supplemented with selenite (≤ 1 mg L^−1^), but it was inhibited under high Se and As (5 mg L^−1^). In another work, Se (IV; 10 μM) boosted the growth of primary roots, but reduced the growth of lateral roots. Moreover, in the same work, Se application downregulated the expressions of genes of auxin and ethylene biosynthesis, and reduced the levels of these hormones in rice roots (Malheiros et al., 2019). Moulick et al. (2016) evaluated the influence of Se-based seed priming in rice under As stress and reported improved germination by 9%, and shoot-, root-length, and plant biomass by 1.6, 1.3, and 1.4-fold, respectively compared with non-treated As stressed plants.

Alves et al. (2020) demonstrated that Se improved photosynthesis and caused alteration in anatomical traits. In *B. napus*, Se application induced reduction in phytotoxicity, resulting in enhanced growth, biomass, photosynthetic pigments, leaf gas exchange, and F_v_/F_m_ (Ulhassan et al., 2019). Khan et al. (2015) reported a decrease in Cd-induced oxidative stress following the application of Se in wheat plants. Sun et al. (2016) demonstrated that Se application caused a reduction in Cd-induced phytotoxic effects on cucumber plants by regulating stress response related proteins and pathways, such as glycolysis pathway and nitrate assimilation pathway (i.e., fructose bisphosphate aldolase 2, NiR), which may increase Se-induced Cd tolerance. Chauhan et al. (2020) reported a boosted activity of glutathione *S*-transferases (GST), peroxiredoxin, glutaredoxins, and heat shock proteins resulting in amelioration of As-induced oxidative stress in rice. In another work, combined application of 24-epibrassinolide (EBL) and Se under Cu stress ameliorated the negative impacts of Cu in *B. juncea via* altering Pro metabolism with increasing Pro accumulation (87%) through stimulating proline synthesizing enzymatic activity of pyrroline-5-carboxylate synthase (Yusuf et al., 2016). In addition, foliar application of both Se and EBL caused the higher activities of RuBisCO and carbonic anhydrase and also improved net photosynthetic rate and stomatal conductance under Cu toxicity.

### Se Improves Ion Homeostasis Under Metal/Metalloid Toxicity

Although studies related to ion homeostasis in response to Se application under heavy metal stress are available, but the available ones have shown positive results regarding said trait in plants. For instance, Zhao et al. (2019) examined the impact of Se supplementation in in *B. campestris* sp. *Pekinensis* grown under Cr stress and observed that 0.1 mg L^−1^ of Se-induced enhanced uptake of nutrients including Na by 40%, Ca by 32%, Fe by 63%, Mn by 21%, Cu by 24%, and Zn by 30% under 1 mg L^−1^ of Cr stress due to alterations in root morphology. In another work, Ulhassan et al. (2019) tested the effect of 5 and 10 μM of Se supplementation on 100 and 200 μM of Cr in *B. napus*. The authors noted a decline in major (N, P, K) and micronutrients (Zn, Fe, Mn) uptake under Cr stress. Application of Se (5 and 10 μM) notably improved the mineral transportation under Cr contamination condition which was restricted in only stress condition (Ulhassan et al., 2019). The above discussed studies demonstrates the positive impact of Se application on ion homeostasis under heavy metals stress (Table 1). However, more research is needed to unveil the mechanisms involved in Se induced maintaining of ions homeostasis in plants.

### Se Enhances Antioxidant Defense Under Metal/Metalloid Toxicity

**Figure 2:**
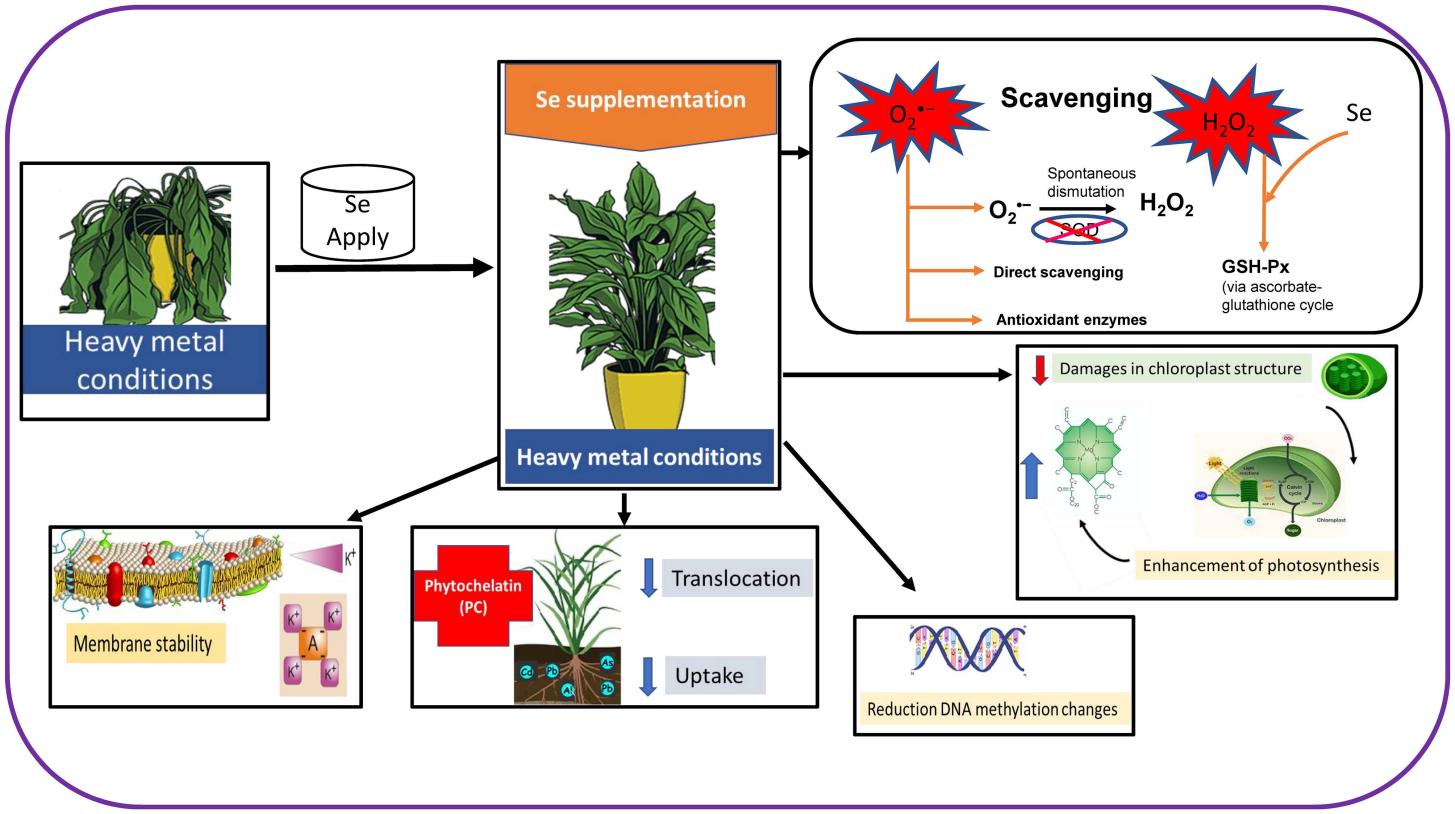
Effects of Se supplementation in plants under heavy metal conditions.

The Se application in stressed plants results in a decrease in ROS *viz*. OH^•^, ${\text{O}}_{2}^{•-}$, and H_2_O_2_ levels, which protects the plants from oxidative stress (Hasanuzzaman et al., 2020). One of the main functions of Se is the enhancement of GPX activity because selenocysteine is present at the catalytic site of this enzyme. Under metal/metalloids stress, plants suffer several damages in different organelles like chloroplast, which suffer modifications in their shape and organization. Nevertheless, the supply of appropriate levels of Se reduces the level of damages in chloroplast and enhances the photosynthetic process, which has been disrupted (Feng et al., 2013). There are several works reporting the positive effect of Se application in the photosynthetic apparatus (Chauhan et al., 2017), which can be associated with a higher capacity of Fe uptake and which have a crucial role in the electron transport chain (ETC), resulting in the generation of substrates to remain organized under high excitations of electronic levels (Feng et al., 2013). Moreover, Se is involved in a higher synthesis of PC or the generation of nontoxic Se metal complexes, which are responsible for the reduction of metals/metalloids resulting in toxicity (Hawrylak-Nowak et al., 2014). The Se application also has a positive effect in DNA structure avoiding methylation changes, which occurs in species grown under metal stress conditions (Figure 2; Filek et al., 2008).

Ulhassan et al. (2019) noted a reduction in the generation of ROS species such as H_2_O_2_, ${\text{O}}_{2}^{•-}$, and MDA, a higher membrane integrity capacity associated with the increase of GSH and AsA levels, and lower activities of SOD and CAT and enzymes involved in AsA–GSH cycle (ascorbate peroxidase, APX; monodehydroascorbate reductase, MDHAR; dehydroascorbate reductase, DHAR; and glutathione reductase, GR) under Cr stress. Moreover, the application of Se also resulted in a reduction of glyoxalase I (Gly I) and glyoxalase II (Gly II) enzyme activities, and consequently in a decline of MG contents. Hawrylak-Nowak et al. (2014) reported decrease in MDA in Se-treated cucumber plants. Qutab et al. (2017) demonstrated that Se supplementation to root system reduced Cd stress triggering a decrease in protein concentrations in the roots of maize exposed to different Cd levels. Sun et al. (2013) found that the application of exogenous Se reduced the toxicity to the different Cd levels. Similarly, in Cd-stressed rice, supply of Se significantly reduced Cd accumulation in rice grains (Hu et al., 2013). Wu et al. (2020) investigated the combined impact of Se and Zn on wheat grown under Cd stress. They observed an increase in photosynthesis and antioxidant enzyme activity, but a decrease in MDA in plant tissues. Zhang et al. (2019) discerned the interaction of different sites with natural presence of Se and Cd and reported that the high ratio of Se to Cd in soil has a beneficial effect on mitigation of Cd stress and its uptake in maize plant.

Reviewing previous literature, there are several references focused on the ameliorative effects of the supply of Se on the antioxidant defense of different species grown under metal/metalloid toxicity (Table 2). The effects of increasing supplies of Se as Na_2_SeO_4_ amendments (0, 2, 4 and 6 μM kg^−1^) in *B. juncea* plants were tested in a pot experiment with soils containing increasing Cr concentrations (0 and 300 μM kg^−1^). The levels of H_2_O_2_ and ${\text{O}}_{2}^{•-}$ were reduced and there was a significant increase in SOD, CAT, MDHAR, DHAR, GR, and GPX activities as well as in non-enzymatic antioxidant *viz*. AsA and GSH due to the external supply of Se (Handa et al., 2019).

**Table 2:** Effects of selenium application on the antioxidant defense in several species under metal/metalloid toxicity.

**Species**	**Doses of selenium**	**Mode of application**	**Metal/metalloid doses**	**Antioxidant defense**	**References**
*Lycopersicum escullentum*	0, 1, 5 and 10 μM of Na_2_SeO_3_	Diluted in hydroponic solution	Cd; 0.5 mM CdCl_2_	Decrease in lipid peroxidation and reduction in SOD, CAT and GR activities	Alves et al., 2017
*L. escullentum*	10 μM of Na_2_SeO_3_	Diluted in hydroponic solution	Cd; 150 mg L^−1^ CdSO_4_·8H_2_O	SOD, CAT, APX and GR activities increased	Alyemeni et al., 2018
*Triticum aestivum*	0.4 and 0.8 mg Se^6+^ kg^−1^ soil	Supplied in soil	Pb; 50 and 100 mg Pb^2+^ kg^−1^ soil	GR and GPX activities increased	Balakhnina and Nadezhkina, 2017
*Brassica napus*	0, 1, 5, 10, 15, 20 mg kg^−1^ soil	Supplied in the substrate	Pb; 300 or 500 mg kg^−1^	SOD and GPX activities decreased	Wu et al., 2016
*B. napus*	50 and 100 μM as Na_2_SeO_4_	Diluted in hydroponic solution	Cd; 0.5 and 1.0 mM CdCl_2_	APX, GR and GPX activities increased	Hasanuzzaman et al., 2012
*Capsicum frutescence*	0, 3 and 7 μM Na_2_SeO_3_	Diluted in hydroponic solution	Cd; 0.25 and 0.5 mM CdCl_2_	POD and CAT activities increased	Shekari et al., 2017
*Raphanus sativus*	2, 4 and 8 mg L^−1^ Na_2_SeO_3_	Diluted in hydroponic solution	Cd; 5, and 10 mg L^−1^ Cd SO_4_	Lipid peroxidation reduced and GPX, CAT, APX activities increased	Amirabad et al., 2020
*Cucumis sativus*	0, 5 or 10 μM as Na_2_SeO_4_	Diluted in hydroponic solution	Cd; 25 or 50 μM, as CdCl_2_·2.5 H_2_O	Lipid peroxidation reduction	Hawrylak-Nowak et al., 2014
*Oryza sativa*	(0.0, 5, 10, 25 and 50 μM Na_2_SeO_3_	Diluted in hydroponic solution	As; 25 μM sodium arsenite (Na_2_HAsO_2_)	APX, CAT and GPX activities increased	Kumar et al., 2014
*R. sativus*	1, 3, 6, 12, and 24 mg Na_2_SeO_4_ kg^−1^ soil	Supplied in the substrate	As; 30 mg As(III) kg^−1^ soil	APX, GR and DHAR activities increased	Hu L. et al., 2020
*Phaseolus aureus*	2.5, 5.0 μM Na_2_SeO_4_	Diluted in hydroponic solution	As; 2.5, 5.0, 10 μM	SOD, CAT, and GR activities increased	Malik et al., 2012
*B. juncea*	Se (50 μM)	Diluted in hydroponic solution	Cd; 100. and 200 mg L^−1^ CdSO_4_·8H_2_O	SOD, APX and GR activities increased	Ahmad et al., 2016

There are also studies about the ameliorative effects of Se in several species under Cd stress. The foliar spraying of Se (Na_2_SeO_4_) in the different growth stages in wheat plants grown under Cd-contaminated soil resulted in higher biomass accumulation and antioxidant enzyme activity. Lipid peroxidation decreased whereas leaf SOD, POD, CAT, and APX increased significantly with the supplies of Se (Wu et al., 2020). Presoaking sunflower seeds with Se (5, 10, and 20 μM) resulted in a reduction of the oxidative damage associated with Cd (20 μM of Cd) toxicity. Presoaking with Se induced an enhancement of CAT, APX, and GR activities (Saidi et al., 2014). Two varieties of wheat (soft and durum) showed a reduction of Cd toxicity due to the external supply of Se *via* root and foliar, where a reduction of lipid peroxidation and an increase in SOD and POD activities were observed (Zhou et al., 2021).

Regarding As toxicity, Singh et al. (2018) assessed the effects of Se application in rice plants. The chemical analysis reported an antagonism between both nutrients reducing the uptake of As under increasing Se concentrations. This antagonism also resulted in lower values of MDA concentration as well as the consequent rise of antioxidant enzymes such as SOD, CAT, and APX. Similarly, in wheat plants the supply of 10 μM of selenate in the nutrient solution resulted in lower oxidative damage in seedlings exposed to increasing As concentrations. Selenium has improved the growth of wheat plants, thereby increasing the activity of APX as well as GPX and GR (Ghosh and Biswas, 2017). In case of rice plants, Pandey and Gupta (2018) reported a clear reduction in the accumulation of H_2_O_2_ as well as ${\text{O}}_{2}^{•-}$ and the increase in the activity of several antioxidant enzymes (CAT, SOD, POD, and GR) with an exogenous supply of Se in rice plants under As stress.

The damages caused by Pb stress can be ameliorated by external supplies of Se. For instance, Mroczek-Zdyrska and Wójcik (2012) reported that the supply of Se at concentrations of 1.5 and 6 μM as sodium selenite in a Hoagland nutrient solution alleviated Pb toxicity (50 μM) in *V. faba* roots. The alleviation was based on the reduction of ${\text{O}}_{2}^{•-}$ generation in the apical parts of roots and enhanced the total sulfhydryl content and GPX activity. Moreover, the supply at low dosage enhanced cell viability, whereas at high levels it improved both lipid peroxidation and cell membrane injury. Zhou et al. (2017) investigated the positive role of Se in alleviating Hg stress. The supplies of increasing concentrations of Se (1, 2, 3, and 4 μM) under 0.5 mg L^−1^ Hg stress resulted in enhanced antioxidant activities (SOD, POD, CAT, and APX). Consequently, there is an increase in the scavenging of ROS and a reduction in lipid peroxidation.

Dai et al. (2019) assessed the positive role of Se in *B. campestris* sp. *Pekinensis* grown under Zn stress. They worked with two treatments (0.5 mg kg^−1^ Se + 30 mg kg^−1^ Zn and 1.0 mg kg^−1^ Se + 30 mg kg^−1^ Zn), and the data obtained reported that the antioxidant enzyme activities increased (POD, SOD, CAT, APX, GR) as well as Pro concentration under Se supplies. Cartes et al. (2010) tested the effects of Se supplies in ryegrass grown under Al stress in hydroponic conditions. They applied 0.2 mM Al and six levels of Se (Na_2_SeO_3_·5H_2_O) (0, 1.0, 1.5, 2.0, 5.0, and 10 μM) for 20 days. Selenium application increased POD activity whereas SOD showed a significant decrease. Hawrylak-Nowak and Matraszek-Gawron (2020) tested if there were differences between selenite and selenate application in lettuce exposed to Ni stress for 14 days. The data reported that selenate had a higher ameliorative effect compared with selenite and also a dose-dependent response. Zhu and Ma (2018) studied the influence of sodium selenite (Na_2_SeO_3_) on physiological characteristics of wine grape seedlings under Cu stress in a greenhouse pot experiment. Selenium treatment (1.0 mg kg^−1^ soil) enhanced pigment concentration, POD, and CAT activities and reduced the level of lipid peroxidation.

### Se-induced Yield Improvement in Plant Under Metal/Metalloid Toxicity

With respect to yield, only limited number of studies have demonstrated the yield potential under Se-supplemented heavy metal stressed crop plants. Shekari et al. (2019) evaluated the impact of Se supplemented at different growth stages on cucumber (*Cucumis sativus* L.) grown under Cd and Pb stress. The authors observed that 4 and 6 mg L^−1^ of Se application induced an increase in flowering, reduction in flowering time, improved female to male flowers ratio, and induced significant enhancement in fruit yield and production time under 60 mM Pb and 100 mM Cd stress compared with the non-treated heavy metals stressed plants (Shekari et al., 2019). In a pot experiment under greenhouse conditions, Huang et al. (2017) tested the effect of Se application in three rice cultivars (Zhongjiu A (female parent) and Huazhan R (male parent), and their F1 hybrid) grown under Cd stress and observed that Se enhanced dry weight of the male parent of rice grain and F1 hybrid in a concentration-dependent manner at the same level of Cd stress. Mozafariyan et al. (2014) examined the impact of Se (0, 3 or 7 μM) under Cd (0, 0.25 or 0.50 mM) on peppers. The authors noted that under Cd stress (0.25 mM), Se applications at 3 or 7 μM while at 0.5 mM Cd level, 3 μM of Se enhanced fruit yield per plant through increasing photosynthetic pigments and improving antioxidant (Mozafariyan et al., 2014).

## Conclusion and Outlook

It is now quite obvious that metal toxicity is not only harmful for plant growth, development, and yield but also a threat for human health as metal enters the human body *via* the food chain. Therefore, consequences of heavy metal stress to agriculture and environment are very alarming and hence demands great attention to search and develop approaches for plant tolerance. Currently, exogenous Se application has been implemented as a remedial strategy for managing metal toxicity because of its beneficial functions. In this review, Se-induced mechanisms in inhibiting the metal stress in plant, including reduction of metal uptake, and translocation to aerial parts, redistribution of subcellular metal distribution such as chelation and compartmentalization, improvement in plant nutrition as well as recovering photosynthesis and maintenance of osmoregulation, are discussed. Upon exposure to metal stress, Se induced the upregulation of antioxidants responses, lowering ROS generation in plant along with strengthening of cell membrane stability. Use of Se for increasing yield under metal contamination has been also demonstrated, which resulted in better production in plants and thus provides new hope to increase food production under the threat of heavy metals. However, for practical utilization, it is imperative to test optimal range of Se for a specific plant species in a particular growing media as the optimal range of Se is quite narrow. The functionality of Se also varies with soil type, treatment time, method of application, and experimental conditions. Testing of all these factors for attaining the best potential of Se against heavy metals is still at an infancy stage and therefore requires future research attention, especially under field conditions. Research must also be executed from a safety view point, as broad scale Se application in agriculture sector, especially at high concentration, may cause toxicity in plants and animals or even higher intake of Se may induce diseases in humans (Rizwan et al., 2020). From the discussion, the positive impacts of Se for increasing plant tolerance under metal stress are obvious. Not only that, more researches in including the supplemental Se effects on food value needs to be executed by which human health hazardous issues could be minimized. In addition, the interactive effects of Se to other metabolites, plant nutrients, hormone, and signaling molecules should be emphasized by in-depth studies for getting best approaches in increasing plant tolerance to metal toxicity with the reduction of residual effects in food crops. However, in-depth studies considering Se-induced mechanisms in plant at molecular and physiological levels need to be investigated further in field condition for economically important plant species.

## Author Contributions

MH conceived the idea, prepared the outline of the manuscript, guided the writing process, revised, and edited the manuscript. All authors have collected the literature, written the manuscript draft, read, and agreed to the published version of the manuscript.

## Conflict of Interest

The authors declare that the research was conducted in the absence of any commercial or financial relationships that could be construed as a potential conflict of interest.

## Publisher's Note

All claims expressed in this article are solely those of the authors and do not necessarily represent those of their affiliated organizations, or those of the publisher, the editors and the reviewers. Any product that may be evaluated in this article, or claim that may be made by its manufacturer, is not guaranteed or endorsed by the publisher.
